# Resting site use of giant pandas in Wanglang Nature Reserve

**DOI:** 10.1038/s41598-017-14315-x

**Published:** 2017-10-23

**Authors:** Dongwei Kang, Xiaorong Wang, Junqing Li

**Affiliations:** 1 College of Forestry, Beijing Forestry University, Beijing, 100083 China; 2 Wanglang National Nature Reserve Administration Bureau, Mianyang, 622550 China

## Abstract

Little is known about the resting sites used by the giant panda (*Ailuropoda melanoleuca*), which restricts our understanding of their resting habits and limits conservation efforts. To enhance our understanding of resting site requirements and factors affecting the resting time of giant pandas, we investigated the characteristics of resting sites in the Wanglang Nature Reserve, Sichuan Province, China. The results indicated that the resting sites of giant pandas were characterised by a mean slope of 21°, mean nearest tree size of 53.75 cm, mean nearest shrub size of 2.82 cm, and mean nearest bamboo number of 56. We found that the resting sites were closer to bamboo than to trees and shrubs, suggesting that the resting site use of giant pandas is closely related to the presence of bamboo. Considering that giant pandas typically rest near a large-sized tree, protection of large trees in the forests is of considerable importance for the conservation of this species. Furthermore, slope was found to be an important factor affecting the resting time of giant pandas, as they tended to rest for a relatively longer time in sites with a smaller degree of slope.

## Introduction

The giant panda (*Ailuropoda melanoleuca*) is a rare and endangered species, which is mainly distributed in the Sichuan, Shaanxi, and Gansu provinces of China^1^. According to the latest report released by the State Forestry Administration of China, the population size of wild giant pandas is now 1864, but is divided into 33 small populations because of natural segregation and human disturbance^2^. Furthermore, habitat fragmentation is still a major threat to the survival of the giant panda^2–4^. Giant pandas continue to face the risk of extinction, and there is still a long way to go to ensure the protection of this species.

A complete understanding of habitat requirements is very important for giant panda protection^5^ because it can provide a scientific basis for habitat restoration, planning, and management^6–8^. Through many years of research efforts, an increasing amount information regarding giant panda habitats has been accumulated, including that on habitat selection^5,9–11^, habitat use^12–14^, habitat preference^15–17^, habitat assessment^18–20^, the effect of human factors^21–24^, climate change^25–27^, habitat restoration^28–31^, etc^32,33^. This information is very useful and has played an important role in giant panda protection. However, it is not sufficient for effective conservation because there are still some topics on which we have little knowledge. For example, some studies have reported that giant pandas often rest at sites where they can lean^34^ and have described resting behaviours such as posture^34^, defaecation^34,35^, and resting time^34,35^. However, there has been little systematic and quantitative research on the resting site characteristics of the giant panda, which restricts our understanding on resting habits and limits our ability to provide effective protection. Thus, we urgently need to carry out relevant research to enhance our understanding of the habitat requirements of the giant panda.

Unlike an ordinary habitat, it is difficult to locate giant panda resting sites in the field, which may be an important reason for the lack of related reports. Consequently, an exploratory study on resting site use is needed to accumulate experience and knowledge, although in this regard there is little published information for reference. In this study, we attempted to characterise the giant panda’s requirements for resting sites based on the use of resting sites in the Wanglang Nature Reserve, Sichuan Province, China. Our main objective was to investigate the characteristics of resting sites in order to determine the factors affecting the resting time of giant pandas. We were particularly interested in the slope of the habitat and the nature of the surrounding vegetation (trees, shrubs, and bamboo) because these are important factors affecting the habitat use of the giant panda^12,14,30,36,37^. We hope this timely report will provide basic data for understanding the resting habits of giant pandas and serve as an important reference for giant panda protection.

## Results

### Resting site characteristics

The resting sites of giant pandas in Wanglang were characterised by a mean slope of 21 ± 8°, mean nearest tree size of 53.75 ± 26.03 cm, mean nearest shrub size of 2.82 ± 2.43 cm, and mean nearest bamboo number of 56 ± 31. The mean distances to the nearest tree, shrub, and bamboo were 1.18 ± 0.91 m, 1.15 ± 0.81 m, and 0.39 ± 0.57 m, respectively.

There was a significant difference among the distances to the nearest tree, shrub, and bamboo (*χ* ^*2*^ = 16.30, *df* = 2, *P < *0.01). The distance to the nearest bamboo was significantly shorter than that to the nearest tree (*t* = −4.52, *P* < 0.01) and the nearest shrub (*t* = −4.66, *P* < 0.01); however, there was no significant difference between the distance to the nearest tree and nearest shrub (*t = *−0.14, *P* > 0.05). Furthermore, 94.9% (37 of 39) of the resting site plots contained trees, and 75.7% (28 of 37) of these trees had a diameter at breast height (DBH) of more than 30 cm (trees with a DBH greater than 30 cm were defined as large-sized trees in this study).

### Resting time prediction

Of the seven variables selected, only slope differed significantly between long-time and short-time groups (*P* < 0.05; Table 1). Compared with the short-time resting sites, the long-time resting sites were characterised by a more gentle slope (Table 1).

**Table 1:** Mean (SD) or mean rank, ANOVA, and *U* test for variables in short-time and long-time groups.

Variable	Mean (SD) or mean rank		F or Z value	P value
	Short-time group (n = 19)	Long-time group (n = 20)		
Slope	24 (8)	18 (8)	5.24	0.03
Nearest tree size	58.05 (27.53)	49.67 (24.56)	0.96	0.33
Nearest tree distance	1.24 (0.88)	1.13 (0.96)	0.15	0.71
Nearest shrub size	17.41	19.38	−0.56	0.58
Nearest shrub distance	1.21 (0.94)	1.10 (0.71)	0.15	0.70
Nearest bamboo number	47 (31)	65 (30)	3.52	0.07
Nearest bamboo distance	19.58	20.40	−0.24	0.84

Logistic regression analysis revealed that none of the absolute correlation coefficients among these seven variables were greater than 0.5 (Table 2), and, furthermore, only slope contributed significantly to the difference between long-time and short-time groups (*P* < 0.05; Table 3). The overall correct prediction rate was 70.6% (Table 4).

**Table 2:** Pearson’s correlation coefficients between 7 variables for all plots.

Variable	Slope	Nearest tree size	Nearest tree distance	Nearest shrub size	Nearest shrub distance	Nearest bamboo number	Nearest bamboo distance
Slope	1	−0.09	0.14	0.14	0.12	−0.09	0.25
Nearest tree size		1	−0.29	0.06	0.08	−0.17	0.06
Nearest tree distance			1	−0.03	−0.10	−0.04	0.08
Nearest shrub size				1	−0.14	−0.05	−0.04
Nearest shrub distance					1	0.10	0.10
Nearest bamboo number						1	0.12
Nearest bamboo distance							1

**Table 3:** Results of logistic regression analysis (using enter method).

Variable	Regression coefficient	Wals	P value
Slope	−0.18	6.22	0.01
Nearest tree size	−0.03	1.66	0.20
Nearest tree distance	−0.18	0.10	0.75
Nearest shrub size	0.17	0.83	0.36
Nearest shrub distance	−0.20	0.07	0.79
Nearest bamboo number	0.02	1.23	0.27
Nearest bamboo distance	0.57	0.58	0.45
Constant	4.48	3.01	0.08

**Table 4:** Prediction results of logistic regression analysis.

Category	Predicted		Percentage correct (%)
	Short-time group	Long-time group	
Short-time group	10	5	66.7
Long-time group	5	14	73.7
Overall percentage (%)			70.6

## Discussion

For giant pandas, bamboo is virtually the sole food source. However, bamboo has low nutritional value and the lignin and cellulose in bamboo are difficult to digest. Consequently, giant pandas normally need to consume large amounts of bamboo to meet their nutritional requirements^34^. For example, it has been reported that a panda can eat from 23 to 38 kg of new shoots per day^34^, and spend more than 50% of the day foraging^38^. Bamboo is therefore vital for giant panda survival. In this study, we found that the resting sites were closer to bamboo than to trees and shrubs, suggesting that the resting site use of giant pandas is closely related to the presence of bamboo. A possible reason for this relationship is that bamboo could provide food for the giant panda after resting and being closer to bamboo could help pandas reduce energy consumption by shortening the distance to the food, which could facilitate more convenient foraging.

According to our previous field surveys, giant pandas normally rest near a large-sized tree, and the results of the present study are consistent with this finding. A possible reason for this is that large trees are more likely to provide resting sites for the giant panda, such as branches and tree holes, as well as coverage under the tree^34,38^. Furthermore, trees with thick trunks could provide a shield and concealment when resting, for example, blocking wind, providing shelter from rain and snow^38^, and even reducing the likelihood of disturbance or attack from other animals^37^. This protection could thus enhance the safety of the giant pandas when resting. In addition, large-sized trees also play important roles in the exchange of information between different individuals, because they are important objects for scent-marking^39^. Thus, we infer that a large tree is an important aspect of a giant panda resting site, and therefore protection of large trees in the forest is of considerable importance for giant panda conservation.

Some authors have assumed that a gentle slope is suitable for feeding^35^ by reducing energy consumption when foraging^40^ or providing easier access to old shoots^36^. These inferences can help us to understand the habitat selection of giant pandas but may not be applicable to resting site use, because giant pandas normally remain motionless or are repeatedly changing postures when resting^34^. We suspect that a resting site with a smaller degree of slope could help the panda fix its body position and keep the body stable when sitting, turning over, or transitioning between postures, which could aid comfort and improve the quality of rest.

This study is an exploratory piece of research. The resting sites of giant pandas are difficult to locate in the field, which is an important reason explaining why the sample size of this study is not large. We found only 39 resting sites in the field, which may be an insufficient number on which to base an explanation of the factors influencing resting site selection by giant pandas. Nevertheless, our findings can serve as basic data for gaining a preliminary understanding of resting habits and behaviour of this species.

In this study, we described the resting site use of giant pandas in the Wanglang Nature Reserve. We found that the resting sites of giant pandas were located at a shorter distance from bamboo than from trees and shrubs, indicating that resting site use in giant pandas is closely related to the presence of bamboo. Giant pandas typically rest near a large-sized tree; therefore, protection of large trees is of considerable importance for the protection of the giant panda. Slope was found to be an important factor affecting the resting time of giant pandas, with individuals tending to rest for a relatively longer time in resting sites with a smaller degree of slope.

## Methods

### Study area

The field survey was carried out in the Wanglang Nature Reserve (103°50ʹ–104°58ʹE, 32°49ʹ–33°02ʹN), which was one of the earliest established giant panda nature reserves in China^38^. The area of this nature reserve is 322.97 km^2^ ^1^. The elevation ranges from 2,320 m to 4,891 m, the average air temperature is −6.1 °C in January and 12.7 °C in July, and the annual rainfall is approximately 862.5 mm^29^. There are 28 giant panda individuals living in this nature reserve^41^, in which the main bamboo species is *Fargesia denudata*.

### Field survey

Giant pandas normally inhabit dense forest environments, and it is difficult to directly observe them and their resting behaviour in the field. The presence of faeces can, however, provide an important reference for identifying resting sites and estimating the resting time of giant pandas^34^. For example, during a short rest of 1 to 2 hours, a giant panda typically discharges 5 to 10 droppings, whereas if the pandas rest for more than 2 hours, they typically discharge at least 10 droppings^34,35^. In this study, we defined a giant panda resting site as a site where a group of more than 10 droppings can be found, and we assumed that a greater number of droppings signifies a longer resting time.

**Figure 1:**
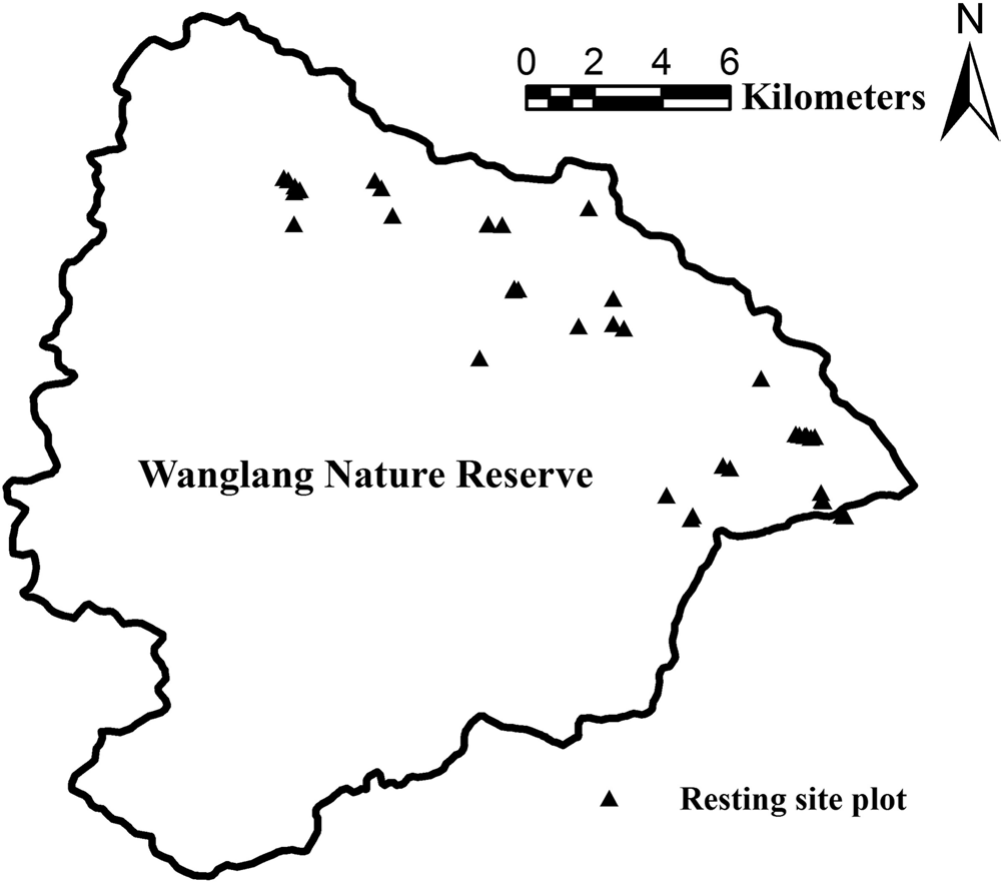
Distribution of resting site plots in this study. This figure was generated by ArcGIS 10.2.

To study the resting site characteristics of giant pandas, we visited the giant panda resting sites that we located in field surveys conducted in April, May, July, August, October, November, and December of 2012^14^; March and April of 2013^30^; and December 2015 in the Wanglang Nature Reserve. A resting site plot was established when a giant panda resting site was encountered in the field and was centralized on the resting site with a size of 5 m × 5 m. Seven variables were measured in each resting site plot (Table 5). We also recorded the number of droppings at each giant panda resting site. In total, 39 resting site plots were sampled for analysis (Fig. 1).

**Table 5:** Description and definition of variables.

Variable	Description
Slope (°)	The slope degree of 5 m × 5 m plot
Nearest tree size (cm)	Breast diameter of tree nearest to the resting site
Nearest tree distance (m)	Distance of the nearest tree to the resting site
Nearest shrub size (cm)	Breast diameter of shrub nearest to the resting site
Nearest shrub distance (m)	Distance of the nearest shrub to the resting site
Nearest bamboo number	Number of bamboo individuals in the nearest bamboo culm
Nearest bamboo distance (m)	Distance of the nearest bamboo culm to the resting site

### Data analysis

To describe resting site characteristics, we calculated the means and standard deviations of the selected seven variables. To determine the relationship between the resting site use of giant pandas and trees, shrubs, and bamboo, we used a Friedman test to compare the difference in variables of distances to the nearest tree, shrub, and bamboo. If this showed a significant difference, we then used a paired-sample *t* test or Wilcoxon test to detect whether significant differences existed between all values based on the statistical requirements.

To determine the factors affecting the resting time of the giant panda, we initially divided the resting site plots into two groups of short-time and long-time based on the number of droppings in each resting site. Plots with less than 20 droppings were placed in the short-time group and other plots were placed in the long-time group. We then used an analysis of variance (ANOVA) or the Mann–Whitney *U* test to compare differences between variables in the different groups based on the statistical requirements. Finally, we used logistic regression analysis to identify variables to discriminate the short-time and long-time groups. To ensure the independence of all variables, we only retained the variable with clear biological meaning if the absolute correlation coefficient of two variables was greater than 0.5^36^.

The significance level of this study was set at 0.05.

## References

[1] State Forestry Administration. *The 3rd National Survey Report on Giant Panda in China* 26–29, 303 (Science Press, 2006).

[2] State Council Information Office of China. The State Forestry Administration held the fourth national giant panda survey results press conference. www.scio.gov.cn/xwfbh/gbwxwfbh/fbh/Document/1395514/1395514.htm (2015) (Date of access: 01/04/2015).

[3] Kang D, Li J (2016). Connect the fragmented habitat patches for giant panda. Environ. Sci. Pollut. Res..

[4] Kang D, Li J (2016). Premature downgrade of panda’s status. Science.

[5] Kang, D. *Research on the Habitat Selection of Giant Pandas* (Dissertation, Beijing Forestry University, 2015).

[6] Ouyang ZY (2002). The recovery processes of giant panda habitat in Wolong Nature Reserve, Sichuan, China. Acta Ecol. Sin..

[7] Shen G (2008). Proposed conservation landscape for giant pandas in the Minshan Mountains, China. Conserv. Biol..

[8] Swaisgood RR (2011). Can science save the giant panda (*Ailuropoda melanoleuca*)? Unifying science and policy in an adaptive management paradigm. Integr. Zool..

[9] Liu X (2005). Giant panda habitat selection in Foping Nature Reserve, China. J. Wildlife Manage..

[10] Yang CH, Zhang HM, Zhou XP, Wang PY, Wang XM (2006). Review of habitat selection in the giant panda (*Ailuropoda melanoleuca*). Acta Ecol. Sin..

[11] Hull V (2014). A synthesis of giant panda habitat selection. Ursus.

[12] Wei F, Feng Z, Wang Z, Hu J (2000). Habitat use and separation between the giant panda and the red panda. J. Mammal..

[13] Lu X, Jiang Z, Li C (2007). Comparative habitat use by giant panda, *Ailuropoda melanoleuca* in selectively logged forests and timber plantations. Folia Zool..

[14] Kang DW, Yang HW, Li JQ, Chen YP, Zhao LJ (2013). Habitat use by giant pandas *Ailuropoda melanoleuca* in the Wanglang Nature Reserve, Sichuan, China. Zool. Stud..

[15] Ran JH (2003). Habitat selection by giant pandas and grazing livestock in the Xiaoxiangling Mountains of Sichuan Province. Acta Ecol. Sin..

[16] Qi D (2011). Different habitat preferences of male and female giant pandas. J. Zool..

[17] Kang DW (2011). Habitat selection attributes of giant panda. Chinese J. Appl. Ecol..

[18] Ouyang ZY, Liu JG, Xiao H, Tan YC, Zhang HM (2001). An assessment of giant panda habitat in Wolong Nature Reserve. Acta Ecol. Sin..

[19] Liu X, Skidmore AK, Bronsveld MC (2006). Assessment of giant panda habitat based on integration of expert system and neural network. Chinese J. Appl. Ecol..

[20] Wang XZ (2008). The application of ecological-niche factor analysis in giant pandas (*Ailuropoda melanoleuca*) habitat assessment. Acta Ecol. Sin..

[21] Bearera S (2008). Effects of fuelwood collection and timber harvesting on giant panda habitat use. Biol. Conserv..

[22] Hull V (2014). Impact of livestock on giant pandas and their habitat. J. Nat. Conserv..

[23] Kang D, Wang X, Yang H, Duan L, Li J (2014). Habitat use by giant pandas (*Ailuropoda melanoleuca*) in relation to roads in the Wanglang Nature Reserve, People’s Republic of China. Can. J. Zool..

[24] Zhao C (2016). Relationship between human disturbance and endangered giant panda *Ailuropoda melanoleuca* habitat use in the Daxiangling Mountains. Oryx.

[25] Tuanmu MN (2013). Climate-change impacts on understorey bamboo species and giant pandas in China’s Qinling Mountains. Nat. Clim. Change.

[26] Li R (2015). Climate change threatens giant panda protection in the 21st century. Biol. Conserv..

[27] Shen G (2015). Climate change challenges the current conservation strategy for the giant panda. Biol. Conserv..

[28] Shen G, Li J, Ren Y, Ma Y (2002). Indicators for giant panda’s habitat degradation and restoration. J. Beijing Forestry Univ..

[29] Wang MJ, Li JQ (2008). Research on habitat restoration of giant panda after a grave disturbance of earthquake in Wanglang Nature Reserve, Sichuan Province. Acta Ecol. Sin..

[30] Kang D, Wang X, Yang H, Duan L, Li J (2014). Habitat use by giant panda in relation to man-made forest in Wanglang Nature Reserve of China. Environ. Sci. Pollut. Res..

[31] Zhang J (2014). Natural recovery and restoration in giant panda habitat after the Wenchuan earthquake. Forest Ecol. Manag..

[32] Wei F, Zhang Z, Hu J (2011). Research advances and perspectives on the ecology of wild giant pandas. Acta Theriol. Sin..

[33] Wei F (2015). Progress in the ecology and conservation of giant pandas. Conserv. Biol..

[34] Hu, J. *Research on the Giant Panda* 88–115, 132–134 (Shanghai Scientific and Technological Education Publishing House, 2001).

[35] Reid DG, Hu J (1991). Giant panda selection between *Bashania fangiana* bamboo habitats in Wolong Reserve, Sichuan, China. J. Appl. Ecol..

[36] Zhang Z (2009). What determines selection and abandonment of a foraging patch by wild giant pandas (*Ailuropoda melanoleuca*) in winter?. Environ. Sci. Pollut. Res..

[37] Kang D, Yang H, Li J, Chen Y (2013). Can conservation of single surrogate species protect co-occurring species?. Environ. Sci. Pollut. Res..

[38] Zhao, X. *Giant Pandas: Natural Heritage of the Humanity* 56–64, 96, 103 (China Forestry Publishing House, 2006).

[39] Liu G, Wang H, Yin Y (2005). Giant panda’s scent marks and scent mark trees in Wanglang National Nature Reserve, Sichuan. Biodivers. Sci..

[40] Wei FW, Feng ZJ, Wang ZW (1999). Habitat selection by giant pandas and red pandas in Xiangling Mountains. Acta Zool. Sin..

[41] Sichuan Forestry Department. *The Pandas of Sichuan: The 4th Survey Report on Giant Panda in Sichuan Province* 10 (Sichuan Publishing House of Science and Technology, 2015).

